# 1-Propyl-4(5)-Methylimidazole Isomers for Temperature Swing Solvent Extraction

**DOI:** 10.3390/molecules27175583

**Published:** 2022-08-30

**Authors:** Shuai Qian, Lauren M. Ward, Luke S. Rakers, Steven T. Weinman, Jason E. Bara

**Affiliations:** Department of Chemical and Biological Engineering, The University of Alabama, Tuscaloosa, AL 35487, USA

**Keywords:** temperature swing solvent extraction, imidazole, molecular design, solvents, heterocycles, amines, solvent extraction desalination

## Abstract

Temperature swing solvent extraction (TSSE) utilizes an amine solvent with temperature-dependent water solubility to dissolve water at a lower temperature to concentrate or crystallize the brine and the phases are separated. Then, the water in solvent mixture is heated to reduce water solubility and cause phase separation between the solvent and water. The solvent and de-salted water phases are separated, and the regenerated solvent can be recycled. Issues with current TSSE solvents include the high solvent in water solubility and the high solvent volatility. This project used the highly tunable platform molecule imidazole to create two 1-butylimidazole isomers, specifically 1-propyl-4(5)-methylimidazole, to test their effectiveness for TSSE. The imidazoles take in more water than their current state-of-the-art counterparts, but do not desalinate the product water and dissolve in water at higher concentrations. Thus, while imidazoles make intriguing candidates for TSSE, further work is needed to understand how to design imidazoles that will be useful for TSSE applications.

## 1. Introduction

Temperature swing solvent extraction (TSSE), also called solvent extraction desalination (SED), is a desalination technique that requires an organic solvent whose miscibility with water is strongly dependent on temperature [1,2,3,4]. TSSE typically utilizes an amine solvent with temperature-dependent water solubility to dissolve water at a lower temperature to concentrate or crystallize the brine and the phases are separated [2,3]. Then, the solvent mixture is heated to reduce water solubility and cause phase separation between the solvent and water. The solvent and de-salted water phases are separated, and the regenerated solvent can be recycled. TSSE using secondary (2°) and tertiary (3°) amines, such as *N*, *N*-diethylmethylamine and triethylamine, was first introduced in the 1960s [5,6,7,8,9]. However, this technique did not gain much traction at that time due to the significant amount of residual solvent in the final product water. Over the past decade, a number of studies have revived the TSSE technique by utilizing long chain carboxylic acids (e.g., octanoic, decanoic) instead of amines [1,10,11,12,13,14,15,16,17,18,19]. The benefit of using these carboxylic acids is the residual levels of solvent in the final product water are at or below the concentrations of these solvents found in dairy products (i.e., FDA approved levels). However, many of these studies mention that the solubility of water in the solvent needs to be increased for commercial viability and almost all of these studies investigated the potential of carboxylic acids for seawater desalination, not brine concentration or crystallization. Additionally, these carboxylic acids have a higher capacity for water at higher temperatures, thus requiring heating (and therefore more energy) of the entire solvent + brine solution.

Amines have a greater water uptake capacity than carboxylic acids and have the benefit of a higher capacity for water at lower temperatures (less heating energy required). Recently, alternative amines (including diisopropylamine (DIPA) and dipropylamine) have been proposed for use in the TSSE process [2,3,20,21,22,23]. However, these amines still face the same issues identified in the 1960s; too much residual solvent left in the final product water [2,3]. This requires the use of stripping or reverse osmosis (RO) as a final polishing step to further recover solvent and purify the final product water.

Other solvent types, such as dimethyl ether (DME) [24,25] and ionic liquids (ILs) [19,26], have been recently investigated for their potential in TSSE. A computational thermodynamic framework was used to estimate that DME could achieve 51 mol% water recovery in a 1-stage process and 63 mol% in 2-stages [25]. The advantage of DME is a very high volatility which enables its rapid separation from water after water absorption [25]. The common IL [C_2_mim][Tf_2_N] was found to have a much (~10×) higher water yield than decanoic acid, very low amounts of IL in the product water (<4 ppm), and high product water purity (97.5% NaCl rejection) [26], however the cost of ILs remains high (~$1000/kg) [19]. As *N*-alkylimidazoles bear similarities to the amines used for TSSE and are also the precursors to ILs, we became interested in how they might be used for TSSE.

In this study, we tested the hypothesis that isomers of butylimidazole will decrease the solvent-water solubility without loss of water solvation relative to DIPA. Butylimidazole was chosen because we had previously observed that among the series of *N*-alkylimidazoles with increasing chain lengths, it was hydrophobic while 1-propylimidazole exhibited partial water miscibility, which is not desired. Imidazoles offer a highly tunable platform by which to rationally design solvents for TSSE applications. Furthermore, 1-butylimidazole (and its isomers) are very similar to DIPA in terms of the number of carbon atoms and their general miscibility with water. Therefore, the use of imidazole as a substrate allows for a greater degree of molecular design in terms of structures which can be tuned to control the phase behavior with brine solutions. The interaction of imidazoles with solutes/solvents is generally governed by position and size of the functional groups which influence properties such as pK_a_, dipole moment, and H-bonding. It is also well-known that these imidazoles have boiling points > 200 °C (i.e., much lower vapor pressures) than amines such as DIPA and thus present a major advantage in terms of minimizing solvent loss due to volatilization. In preliminary observations, it was found that 1-propyl-4(5)-methylimidazole (a mixture of 4-Me and 5-Me isomers) exhibited a significantly higher water uptake and change in water concentration between room temperature and 70 °C (~135,000 ppm) compared to other imidazole compounds (1-propyl-2-isopropylimidazole had a change of ~38,000 ppm, 1-propyl-2-methylimidazole had a change of ~51,000 ppm, 1,2-diisopropylimidazole had a change of ~49,000 ppm, and 1-butyl-2-ethylimidazole had a change of ~4700 ppm). Therefore, this work is focused on 1-propyl-4(5)-methylimidazole synthesis, characterization, and testing for brine desalination for comparison to DIPA.

## 2. Results and Discussion

### 2.1. Solvent Synthesis

A product containing two isomers (1-propyl-4-methylimidazole and 1-propyl-5-methylimidazole) was synthesized starting from 4-methylimidazole and 1-bromopropane in the presence of NaOH. Relative to our prior work, the purification process was improved, leading to an enhancement in the isolated yield from 61.0% [27] to 77.0% (this work). However, the formation of both the 4-Me isomer and the 5-Me isomer are still inevitable due to the reaction kinetics and the tautomerization of 4-methylimidazole starting material. However, it is possible to obtain a product enriched in either the 4-Me or 5-Me isomer by collecting fractions of the distillate. We are not aware of prior works that the reported the enrichment of either isomer through distillation, and it was previously assumed that the isomers were largely inseparable. This allowed for the investigation of the impact of either the 4-Me or 5-Me isomer in an enriched condition.

### 2.2. Desalination Experiments

Desalination experiments were performed with different compositions of the 4(5)-Me imidazole isomers. Additionally, DIPA was tested for comparison. Initial experiments were performed with an isomer ratio of 37:63 (4-Me:5-Me). After the experiments, it was noticed that the isomer ratio changed to 39% of the 4-Me isomer and 61% of the 5-Me isomer, suggesting the 5-Me isomer is more soluble in water. This led us to test the three isomer ratios shown in Table 1. These ratios were used to determine if one isomer had a greater effect on the desalination performance. A more detailed description of the experiments is explained in Section 3.3.1.

#### 2.2.1. Karl-Fisher Titration of Solvent Phases

**Figure 1:**
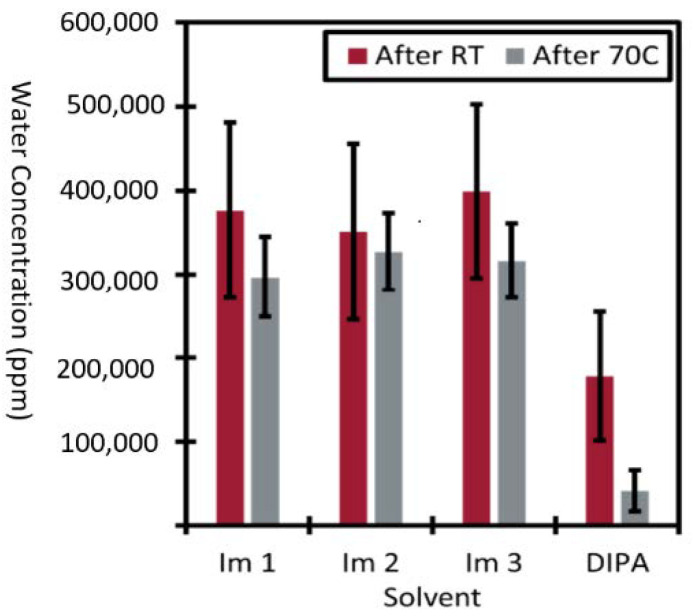
The amount of water present in the solvent after contact with the brine phase and after phase separation at 70 °C as determined by a KF Titrator. The error bars represent one standard deviation among at least three trials.

Approximately 0.4 mL of solvent was removed after each step and saved to be used for Karl-Fisher (KF) Titration to determine how much water was being taken in and released by the solvent. Figure 1 shows the results. The different isomer ratios did not impact the amount of absorbed water at RT, nor the remaining amount of water after 70 °C. Additionally, there was no statistical difference between the amount of water in the solvent at RT and 70 °C even though there appears to be a drop in the average water concentration and there was a measurable amount of water released from the solvent (see Table S1, Table S2, and Table S3 for statistical test information). All imidazole ratios at RT and 70 °C did take in a statistically larger amount of water when compared to DIPA. However, DIPA had a statistical decrease in water concentration in the solvent after phase separation in the hot water bath.

#### 2.2.2. Chloride Ion Concentration of Water Phases

**Figure 2:**
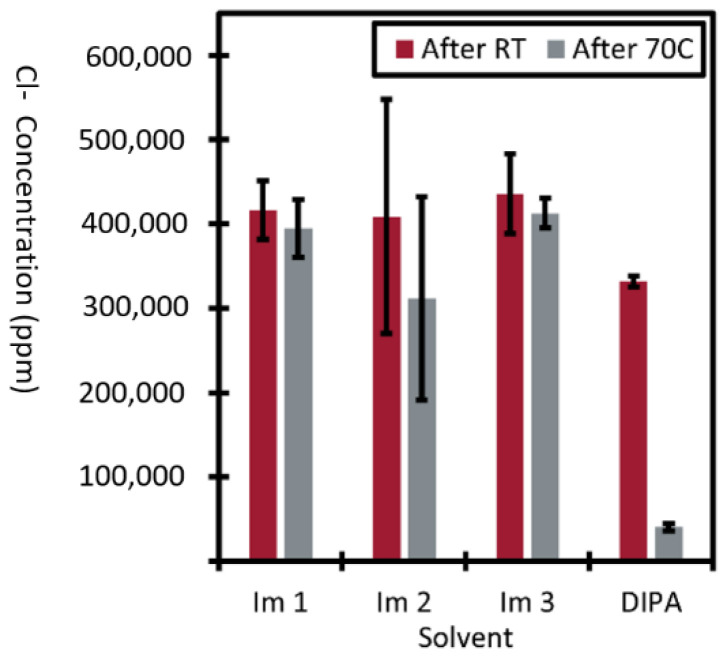
The average amounts of Cl^−^ ion concentration that was in the water phase after contact with the solvent at RT and after release from the solvent at 70 °C. The error bars represent one standard deviation among at least two trials.

Aqueous sodium chloride (NaCl) with a concentration of 100 g/L was used, with an initial Cl^−^ concentration of 60,000 ppm. The water phase that was removed after each step was tested for Cl^−^ concentration. Figure 2 shows that for all the imidazole ratios, the concentration of chloride ions remains approximately the same as the initial brine Cl^−^ concentration after contact with the solvent at RT and after phase separation from the solvent at 70 °C. While it appears there might be a slight decrease in Cl^−^ concentration at 70 °C, the error bars overlap and thus are not statistically different. This indicates that Im 1, Im 2, and Im 3 solvents are not desalinating the product water. However, DIPA shows a significant decrease in ion concentration after phase separation from the solvent at 70 °C from the initial brine phase, indicating reasonable desalination (calculated as ~88% removal). The imidazoles are likely not desalinating the water due to the enveloping of the salt water (i.e., large cavities between solvent molecules) instead of selective water transfer through H- bonding between the imidazoles and water, [22] which has been demonstrated in recent computational results [28]. Alkyl-imidazoles are common ILs because they do not pack well. This poor packing could lead to large cavities between solvent molecules. Future work will explore finding solvents that pack more tightly to provide smaller cavities and force desalination through hydrogen bond formation with water.

#### 2.2.3. UV-Vis of Water Phases

**Figure 3:**
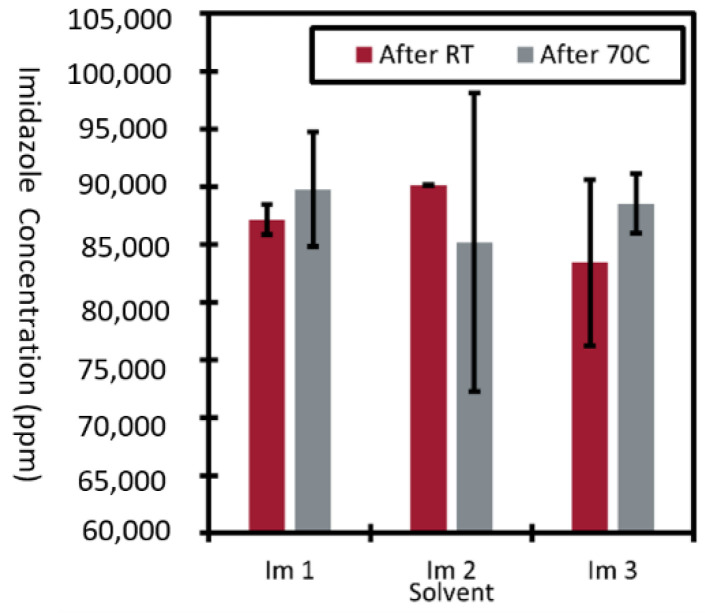
The amount of Im 1, Im 2, and Im 3 solvent that dissolved in the water phase after contact at RT and after phase separation at 70 °C. The error bars represent one standard deviation among at least two trials.

The water phase that was removed after each step also was tested for imidazole concentration after contact with the solvent at RT and after phase separation at 70 °C. Figure 3 shows the amount of Im 1, Im 2, and Im 3 solvent that was dissolved in the water phase. DIPA concentration was not able to be determined due to it not being able to be quantified using UV-Vis. One important note is that UV-Vis was not able to determine the individual isomer ratio that was dissolved in the water phase, so the results are presented as a total imidazole concentration. There is a large amount of imidazole present in the water phases after any contact with the solvent. These values are much larger than the values measured for DIPA in other reports [2,3]. There is no apparent difference between the individual isomer ratios after contact with the solvent at RT and after phase separation at 70 °C. Additionally, there is no apparent difference between the three isomer ratios after contact with the solvent at RT and after phase separation at 70 °C. These results show that the imidazole platform needs to be further explored to find solvents with promise for the TSSE application.

## 3. Materials and Methods

### 3.1. Materials

4-Methylimidazole (>98%) was purchased from TCI America (Portland, OR, U.S.A.); 1-Bromopropane (99%) was purchased from Alfa Aesar (Tewksbury, MA, U.S.A.); ACS grade tetrahydrofuran (THF) was purchased from Macron Fine Chemicals (Radnor, PA, U.S.A.); Dichloromethane (DCM, >99.5%), sodium hydroxide (NaOH, 97%), and sodium bicarbonate (NaHCO_3_, >99.7%) were purchased from VWR (Radnor, PA, U.S.A.); NaCl (≥99.0%, anhydrous) was purchased from Sigma-Aldrich (St. Louis, MO; U.S.A.); DIPA (99%) was purchased from BeanTown Chemical (Hudson, NH, U.S.A.); DMSO-*d_6_* (99% with 0.05% V/V TMS) was purchased from Cambridge Isotope Laboratories (Tewksbury, MA, U.S.A.); Aqueous solutions were prepared with deionized water from a Millipore Synergy^®^ (Burlington, MA, U.S.A.) water purification system.

### 3.2. Imidazole Synthesis and Characterization

#### 3.2.1. Imidazole Synthesis

**Scheme 1:**
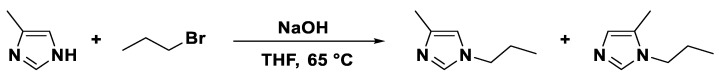
Synthesis method for 1-propyl-4-methylimidazole (left product) and 1-propyl-5-methylimidazole (right product) solvents.

The mixture of 1-propyl-4(5)-methylimidazole was synthesized as shown in Scheme 1 in a similar way we have reported previously [27]. NaOH (90.7 g, 2.2 mol) pellets were loaded into a 1 L round-bottom flask, followed by addition of 4-methylimidazole (92.2 g, 1.1 mol) dissolved in 600 mL THF. The mixture was stirred, heated, and kept at 65 °C for activation for 16 h, after which 1-bromopropane (124.2 g, 1.0 mol) was added dropwise and the reaction was stirred at 65 °C for 24 h. After this time, the reaction was stopped and cooled to room temperature. The solids were filtered and the filtrate was condensed through rotary evaporation. 10 M aqueous NaOH (50 mL) was added to the condensed mixture and stirred for 3 h for deprotonation of the unreacted 4-methylimidazole, DCM (500 mL) was then added to extract the product and separated, followed by washing with aqueous NaHCO_3_ (100 mL) and DI water (100 mL). The organic phase was dried over MgSO_4_, filtered, and reduced through rotary evaporation to give a dark brown crude product. ^1^H NMR of the crude product indicated the absence of residual 4-methylimidazole and majority of organic impurities. The mol% of isomers in this initial product were 60% (4-Me) and 40% (5-Me).

To separate target compounds from inorganic components, a distillation was performed and liquids coming out at different stages were collected and distributed in four flasks using a four-neck distillation receiver (Chemglass CG-1279). Vacuum was applied using a IKA VACSTAR digital pump and the pressure was maintained in the range of 20–40 Torr as indicated by a MKS PDR2000 pressure gauges. A small amount of liquid impurities were collected in the first flask when the liquid mixture was heated to 150 °C while the temperature of vapor was kept below 50 °C. The product was vaporized when heated at 155 °C and the temperature of the vapor was 135 °C. The first ~2 mL product was discarded in flask #1, after which collection of distillate was switched to #2 flask. The liquid mixture was heated to 180 °C with increments of 5 °C to ensure the steady flow of the product, and the liquids were collected in #2–4 flasks in sequence with relatively equal volume. The liquids collected in #2–4 flasks were a total of 95.67 g (77.0%) 1-propyl-4(5)-methylimidazole as a clear, colorless liquid.

#### 3.2.2. Imidazole Characterization

The imidazole compounds were characterized using a Bruker Ascend^TM^ 500 instrument. The pre-distillation mixture showed ^1^H NMR [Figure S1] (500 MHz, DMSO-*d*_6_) δ 7.47 (dd, *J* = 26.6, 1.3 Hz, 1H), 6.72 (dt, *J* = 105.5, 1.1 Hz, 1H), 3.81 (t, *J* = 6.9 Hz, 2H), 2.11 (dd, *J* = 36.1, 1.0 Hz, 3H), 1.66 (dh, *J* = 12.1, 7.3 Hz, 2H), 0.82 (dt, *J* = 16.2, 7.4 Hz, 3H), which agrees well with data reported previously [27]. The mol% of 4-Me and 5-Me isomers for the crude product and liquids collected in different stages of the distillation was analyzed by integration of proton peaks shifted in the range of 6–8 ppm in the ^1^H NMR spectra.

### 3.3. Solvent Performance Evaluation and Characterization

#### 3.3.1. Brine Desalination Testing

20 mL of imidazole and 20 mL of brine (100 g/L NaCl) were mixed together in a separatory funnel. The mixture was shaken for ~1 min and inversed ~5 times to ensure there was full contact between the two phases. The mixture sat at room temperature (~25 °C) for 1 h. Then, the water (bottom) phase was removed and kept for characterization. Additionally, a small amount of solvent (top phase) was removed for characterization. Then, the solvent phase was transferred to a 65 mL test tube and placed in a 70 °C water bath for 1 h, after which the test tube was removed. The temperature of the water bath was controlled using a homemade stainless-steel coil and a Thermo Scientific Accel 500 LC. The solvent (top) phase was removed via pipette for characterization. The separated water (bottom phase) was stored for characterization.

#### 3.3.2. Solvent Characterization

##### Karl-Fischer Titration

Solvent after contact with the brine solution at both room temperature and 70 °C for 1 h was analyzed using a Mettler Toledo C20S Coulometric KF Titrator to determine the water content of the solvent. KF Titration was performed to determine how much water was taken into and separated from the organic phase. Approximately 0.4 mL of the solvent was drawn up in a syringe with a needle attached. The solvent was then injected into the Hydranal™ (Coulomat AG) solvent in the KF titrator.

##### Chloride Ion Concentration

A Mettler Toledo S220 SevenCompact pH/ion meter and a Mettler Toledo PerfectIon Combination Chloride Electrode were used to determine the amount of chloride ion (Cl^−^) present in the water phase. Before taking a reading, a Mettler Toledo perfectION ion strength adjuster (ISA) Solid State solution was used at an amount of 2 mL of ISA per 100 mL of sample. Sample volume was typically less than 10 mL. A calibration curve was made using known concentrations of chloride ions (see Figure S2) to calculate the Cl^−^ concentration of the brine phase after contact with the solvent and the water phase that phase separated out of the solvent.

##### UV-Vis

A HACH DR6000 was used to determine the amount of imidazole that transferred to the water phase. DIPA is not detectable by the UV-Vis. The brine phase after contact with the solvent and the water phase that phase separated out of the solvent was tested for imidazole concentration. A calibration curve was made using known concentrations of imidazole (see Figure S3) to calculate the imidazole concentration based on the absorbance reading. A wavelength scan was done in order to determine that λ = 261 nm was the wavelength that the imidazole responded to and was used for these measurements.

## 4. Conclusions

Three mixtures of 1-propyl-4(5)-methylimidazole isomers were tested for their ability to perform TSSE. The results showed that all three imidazole ratios took in more water than the current state-of-the-art TSSE solvent, DIPA. However, these imidazoles did not remove the salt from the water as determined by chloride probe analysis and these imidazoles dissolved in water at a higher concentration than DIPA as determined by UV-Vis analysis. The inability of these imidazoles to desalinate is likely due to the lack of hydrogen bond formation between the imidazoles and water and large cavities that exist between solvent molecules. Thus, these results disprove our hypothesis that 1-propyl-4(5)-methyl imidazole ratios will decrease the solvent water solubility without loss of water solvation relative to DIPA. However, the imidazole platform is highly tunable and we will continue to investigate, both experimentally and computationally, for other imidazoles that will make TSSE commercially viable, especially imidazoles that can experience H-bonding bond with water. Additionally, we will investigate the compressibility of the solvents with the goal to find solvents with low compressibility, and therefore, smaller cavities to force desalination through hydrogen bond formation with water. Perhaps these solvents could find use in other areas, such as the dehydration of natural gas.

## Figures and Tables

**Table 1:** The different isomer ratios in solvents used for TSEE experiments.

Solvent	% of 1-Propyl-4-Methyl Imidazole	% of 1-Propyl-5-Methyl Imidazole
Im 1	74%	26%
Im 2	65%	35%
Im 3	37%	63%

## Data Availability

The data presented in this study are available on request from the corresponding authors.

## Supplementary Materials

The following supporting information can be downloaded at: https://www.mdpi.com/article/10.3390/molecules27175583/s1, Figure S1. ^1^H NMR spectrum of 1-propyl-4(5)-methylimidazole; Figure S2. Calibration curve and equation for the concentration of chloride ions in a NaCl solution; Figure S3. Calibration curve and equation for the concentration of imidazole that was present in the water phase; Table S1. Results of the two-sample t-test assuming unequal variances for the same ratio/compound; Table S2. Results of the two-sample t-test assuming unequal variances for different ratios/compounds at the RT; Table S3. Results of the two-sample t-test assuming unequal variances for different ratios/compounds at the 70 °C.

## Sample Availability

Samples of the compounds used in this study are not available from the authors due to the limited synthesis scale and the authors’ ongoing need for them in current studies.
