# Supplementary material for: 1-Propyl-4(5)-Methylimidazole Isomers for Temperature Swing Solvent Extraction
Source: Molecules. 2022 Aug 30;27(17):5583. doi: 10.3390/molecules27175583 (PMC9457880; doi:10.3390/molecules27175583)
Supplement: Supplementary file 1 [file molecules-27-05583-s001.zip › molecules-1831116-supplementary.pdf]

1-propyl-4(5)-methylimidazole Isomers for Temperature Swing Solvent Extraction

Shaui Qian[1,†] Lauren M. Ward[1,†], Luke S. Rakers[1], Steven T. Weinman[1,*], Jason E. Bara[1,*]

Department of Chemical and Biological Engineering, The University of Alabama, Tuscaloosa, AL 35487, USA

Supporting Information

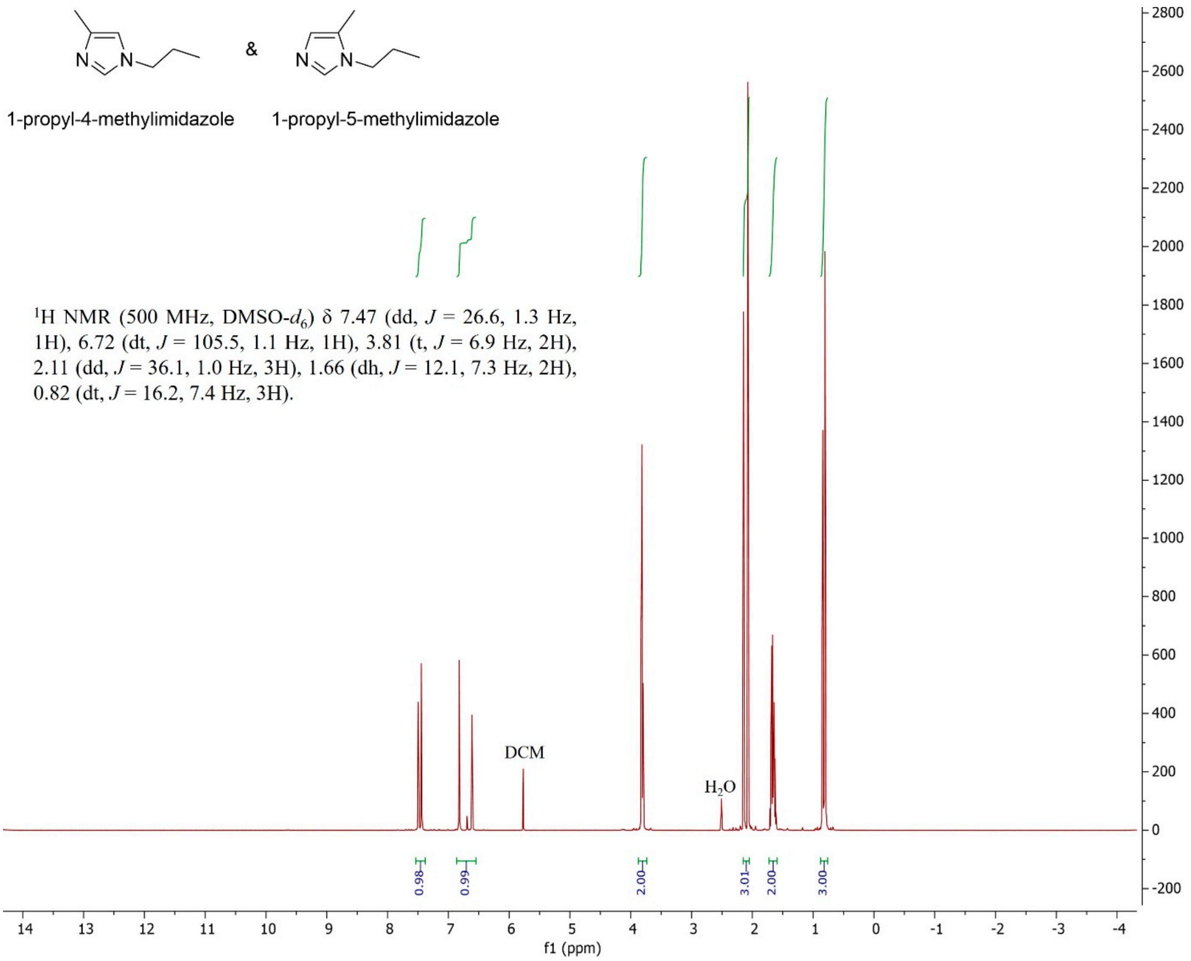

Figure S1. ^{1}H NMR spectrum of initial 1-propyl-4(5)-methylimidazole before distillation.

Calibration for Chloride Concentration

The data and equation present in Figure S2 were used to create a calibration curve of chloride concentration. This was then used to determine the chloride concentration in the samples shown in **Figure 2** in the main document.

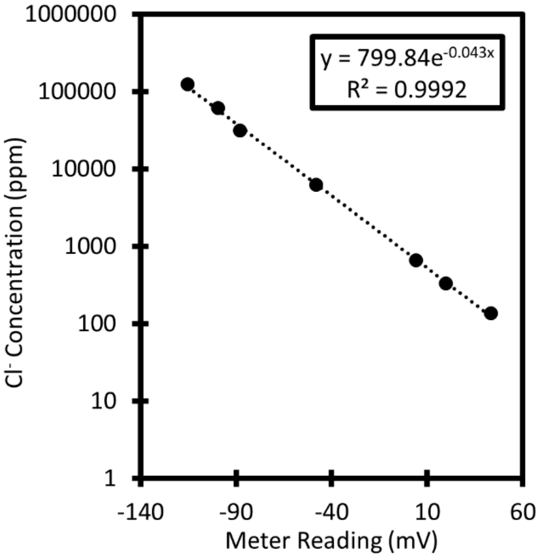

Figure S2. Calibration points and equation of fit (dashed line) for the concentration of chloride ions in an aqueous NaCl solution.

Calibration for Imidazole Present in Water

The data and equation present in Figure S3 were used to create a calibration curve of amount of imidazole in the water phase. This was used to determine the amount of imidazole that transferred to the water phase during experiments that is shown in **Figure 3** in the main document.

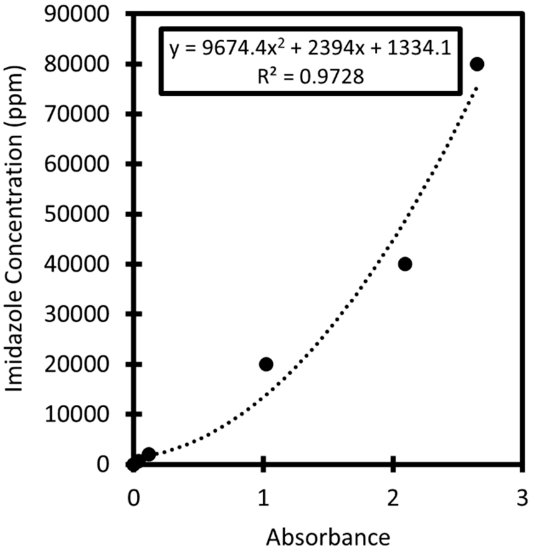

Figure S3. Calibration points and equation of fit (dashed line) for the concentration of imidazole that was present in the water phase.

Results from Two-Sample t-test Assuming Unequal Variances

Hypothesis testing was done to determine the statistical relevance of the data sets. EXCEL (Version) was used for all statistical analyses. All tests were done using 90% confidence ($\alpha = 0.10$); therefore, if the p-value is greater than α then the means are considered to be equal and if the p-value is less than α then the means are considered to be unequal.

The Welch's t-test was used because there was not the same amount of trials between DIPA and imidazole. When comparing the same ratio/compound at different temperatures, a one-tail test was used because the focus was on having a significant decrease in water content. When comparing different ratios/compounds at the same temperature, a two-tail test was used because the focus was on overall differences.

Table S1 shows the results from the statistical tests that were performed with the same ratio/compound but at different temperatures. Table S2 shows the results from the statistical tests that were performed between the different ratios/compounds at RT. Table S3 shows the results from the statistical tests that were performed between the different ratios/compounds at 70 °C. These results were performed on the water concentration data from Figure A in the main document.

Table S1. Results of the two-sample t-test assuming unequal variances for the same ratio/compound.

Different Temperature Comparison					
**Compound**	**Group 1 Data**	**Group 2 Data**	**Test Variable**	**One-Tailed P Value**	**Interpretation (90% Confidence)**
Im 1	RT	70 °C	Water Concentration	0.149	Not statistically significant
Im 2	RT	70 °C	Water Concentration	0.370	Not statistically significant
Im 3	RT	70 °C	Water Concentration	0.135	Not statistically significant
DIPA	RT	70 °C	Water Concentration	0.069	Statistically significant

Table S2. Results of the two-sample t-test assuming unequal variances for different ratios/compounds at the RT.

Comparison at the Same Temperature					
**Temperature**	**Group 1 Data**	**Group 2 Data**	**Test Variable**	**Two-Tailed P Value**	**Interpretation (90% Confidence)**
RT	Im 1	Im 2	Water Concentration	0.776	Not statistically significant
RT	Im 1	Im 3	Water Concentration	0.796	Not statistically significant
RT	Im 1	DIPA	Water Concentration	0.059	Statistically significant
RT	Im 2	Im 3	Water Concentration	0.590	Not statistically significant
RT	Im 2	DIPA	Water Concentration	0.087	Statistically significant

RT	Im 3	DIPA	Water Concentration	0.041	Statistically significant

Table S3. Results of the two-sample t-test assuming unequal variances for different ratios/compounds at the 70 °C.

**Comparison at the Same Temperature**					
**Temperature**	**Group 1 Data**	**Group 2 Data**	**Test Variable**	**Two-Tailed P Value**	**Interpretation (90% Confidence)**
70 °C	Im 1	Im 2	Water Concentration	0.459	Not statistically significant
70 °C	Im 1	Im 3	Water Concentration	0.630	Not statistically significant
70 °C	Im 1	DIPA	Water Concentration	0.000	Statistically significant
70 °C	Im 2	Im 3	Water Concentration	0.772	Not statistically significant
70 °C	Im 2	DIPA	Water Concentration	0.000	Statistically significant
70 °C	Im 3	DIPA	Water Concentration	0.000	Statistically significant
